# GSTO1*CC Genotype (rs4925) Predicts Shorter Survival in Clear Cell Renal Cell Carcinoma Male Patients

**DOI:** 10.3390/cancers11122038

**Published:** 2019-12-17

**Authors:** Tanja Radic, Vesna Coric, Zoran Bukumiric, Marija Pljesa-Ercegovac, Tatjana Djukic, Natasa Avramovic, Marija Matic, Smiljana Mihailovic, Dejan Dragicevic, Zoran Dzamic, Tatjana Simic, Ana Savic-Radojevic

**Affiliations:** 1 Institute of Medical and Clinical Biochemistry, 11000 Belgrade, Serbia; tanjajevtic@gmail.com (T.R.); drcoricvesna@gmail.com (V.C.); m.pljesa.ercegovac@gmail.com (M.P.-E.); tatjana.djukic@med.bg.ac.rs (T.D.); marija_opacic@yahoo.com (M.M.); tatjana.simic@med.bg.ac.rs (T.S.); 2 Faculty of Medicine, University of Belgrade, 11000 Belgrade, Serbia; zoran.bukumiric@med.bg.ac.rs (Z.B.); natasa.avramovic@med.bg.ac.rs (N.A.); dpdragicevic@gmail.com (D.D.); slavica.lisicic@kcs.ac.rs (Z.D.); 3 Institute of Medical Statistics and Informatics, 11000 Belgrade, Serbia; 4 Institute of Medical Chemistry, 11000 Belgrade, Serbia; 5 Clinic for Gynecology and Obstetrics “Narodni front”, 11000 Belgrade, Serbia; smiljanamihailovic@gmail.com; 6 Clinic of Urology, Clinical Center of Serbia, 11000 Belgrade, Serbia; 7 Serbian Academy of Sciences and Arts, 11000 Belgrade, Serbia

**Keywords:** glutathione transferase omega 1, glutathione transferase omega 2, polymorphism, PI3K/Akt/mTOR, Raf/MEK/ERK, IL-1β, pro-IL-1β

## Abstract

Omega class glutathione transferases, GSTO1-1 and GSTO2-2, exhibit different activities involved in regulation of inflammation, apoptosis and redox homeostasis. We investigated the the prognostic significance of *GSTO1* (rs4925) and *GSTO2* (rs156697 and rs2297235) polymorphisms in clear cell renal cell carcinoma (ccRCC) patients. GSTO1-1 and GSTO2-2 expression and phosphorylation status of phosphoinositide 3-kinase (PI3K)/protein kinase B (Akt)/ /mammalian target of rapamycin (mTOR) and Raf/MEK/extracellular signal-regulated kinase (ERK) signaling pathways in non-tumor and tumor ccRCC tissue, as well as possible association of GSTO1-1 with signaling molecules were also assessed. GSTO genotyping was performed by quantitative PCR in 228 ccRCC patients, while expression and immunoprecipitation were analyzed by Western blot in 30 tissue specimens. Shorter survival in male carriers of GSTO1*C/C wild-type genotype compared to the carriers of at least one variant allele was demonstrated (*p* = 0.049). GSTO1*C/C genotype independently predicted higher risk of overall mortality among male ccRCC patients (*p* = 0.037). Increased expression of GSTO1-1 and GSTO2-2 was demonstrated in tumor compared to corresponding non-tumor tissue (*p* = 0.002, *p* = 0.007, respectively), while GSTO1 expression was correlated with interleukin-1β (IL-1β)/pro-interleukin-1β (pro-IL-1β) ratio (*r* = 0.260, *p* = 0.350). Interaction of GSTO1 with downstream effectors of investigated pathways was shown in ccRCC tumor tissue. This study demonstrated significant prognostic role of *GSTO1* polymorphism in ccRCC. Up-regulated GSTO1-1 and GSTO2-2 in tumor tissue might contribute to aberrant ccRCC redox homeostasis.

## 1. Introduction

Being the most common and the most aggressive subtype among renal cancers, clear cell renal cell carcinoma (ccRCC) accounts for most RCC fatal outcomes [1]. Such outcomes probably arise from significant intra-tumor and inter-tumor genetic diversity of ccRCC, revealed in recent genomic studies [1,2,3]. Indeed, among genetic factors that contribute to RCC risk, the most investigated is mutation of *VHL* gene [1], underlying the abnormal accumulation of hypoxia-inducible factor α (HIFα) proteins in normoxia [4]. Namely, downstream over-expression of HIF-targeted genes is involved in the regulation of angiogenesis, proliferation, invasion and survival [4], as well as in the metabolism of glucose, influencing the characteristic metabolic phenotype of the disease [5]. Among the most investigated HIF-targeted genes is vascular endothelial growth factor (VEGF). It binds to specific tyrosine kinase receptor, VEGF-R2, expressed on both endothelial and ccRCC cells [6], further resulting in downstream signaling which mediates the activation of Ras/MEK/ extracellular signal-regulated kinase (ERK) and phosphoinositide 3-kinase (PI3K)/protein kinase B (Akt)/ mammalian target of rapamycin (mTOR) pathway. In this way, tumor progression is promoted by additional HIFα production [5] as a part of positive feedback loop which contributes to constitutive activation of the signaling network [7]. In addition, multiple other mechanisms could contribute to constitutive activation of the PI3K/Akt pathway in ccRCC [7], including epigenetic regulatory mechanisms, specifically microRNAs (miRNAs) [7], as well as protein complex formation with phosphoinositide-dependent kinase-1 (PDK1) and 78-kDa glucose-regulated protein [7]. Interestingly, it has been shown that deglutathionylation type of modification mediated by glutaredoxin 1 was implicated in the activation of Akt, thus, protecting the cells from oxidative stress–induced apoptosis [8].

The role of glutathione transferases (GST) in redox regulation has already been taken into consideration as a contributing mechanism both in cancer development and progression [9,10]. Representing a set of cytosolic, mitochondrial and microsomal proteins with versatile catalytic and noncatalytic functions [11], GSTs have been readily studied in the light of their bio-transformational capacities towards potent xenobiotics, as well as endogenous reactive oxygen species [12]. This may not come as a surprise, since most of the genes encoding for members of GST enzyme superfamily are highly polymorphic, therefore, altering the individual susceptibility to environmental and oxidative stress [9,13]. Additionally, their functional repertoire comprises the ability to form protein-protein interactions, independently of their catalytic functions, thus negatively regulating certain protein kinases involved in cell proliferation and apoptosis [9,13]. In the case of RCC, a growing body of evidence suggests that cytosolic GSTs might be involved not exclusively in the development, but also in the progression of RCC [12,14,15]. However, a couple of studies have tackled the problem of GST polymorphisms with regard to RCC patients’ survival [15,16], proposing the aforementioned protein-protein interactions as the underlying molecular mechanism in RCC progression.

Omega class members, GSTO1-1 and GSTO2-2 isoenzymes, are unique in terms of presence of cysteine in the active site [17], thus, manifesting the whole range of specific activities not associated with other human GSTs [18]. Cysteine residue in the active site allows these isoenzymes to catalyze specific spectrum of glutathione-dependent thiol exchange and reduction reactions [17]. Namely, among other, GST omega class members possess thioltransferase and dehydroascorbate reductase activities, similarly to glutaredoxins [18]. Indeed, GSTO1-1 exhibits deglutathionylase activity [18], and seems to be involved in the modulation of ryanodine receptors, as well as the activation of IL1-β [19,20]. On the other hand, GSTO2-2 is the enzyme with the highest dehydroascorbate-reductase (DHAR) activity, in that way preserving reduced form of ascorbic acid [21]. It has also been shown that in contrast to GSTO2-2, GSTO1-1 plays important role in the glutathionylation cycle by its deglutathionylase and glutathionylase activity, depending on different conditions [22]. Additionally, it has been suggested that GSTO1-1 alters cell survival signaling pathways by inhibiting apoptotic MAPK signaling pathways [23,24].

Recently, novel aspect of GSTO1-1 role in cancer progression is shown to be mediated by interaction with type 1 ryanodine receptor, RyR1 [25]. Namely, Lu et al. showed HIF-dependent expression of GSTO1-1 in breast cancer cells exposed to carboplatin with consequent breast cancer stem cell enrichment, mediated by interaction between GSTO1-1 and RyR1 and downstream activation of PYK2/SRC/STAT3 signaling [25]. Additionally, they demonstrated that GSTO1-1 knockdown blocks cancer stem cell enrichment, tumor initiation and metastasis [25]. The overexpression of GSTO1-1 has been also reported in esophageal squamous cell carcinoma, pancreatic cancer, and ovarian cancer [18]. The study by Piaggi et al. showed that overexpression of GSTO1-1 following cisplatin treatment of HeLa cells seems to be associated with the activation of survival signaling pathways and inhibition of apoptotic MAPK pathway [23].

Based on the association between structure and function, three GSTO polymorphisms seem to be of greatest importance: one transition polymorphism in the position 183 at 5′ untranslated region (5′ UTR) of *GSTO2* gene (GSTO2*A183G, rs2297235), as well as two single nucleotide polymorphisms GSTO1*C419A (rs4925) and GSTO2*A424G (rs156697). *GSTO1* rs4925 polymorphism, causing alanine to aspartate substitution in amino acid 140 (*Ala140Asp), results in a change in its deglutathionylase activity. Namely, GSTO1*C wild-type allele exhibits higher deglutathionylase activity and lower activity in the forward glutathionylation reaction in contrast to GSTO1*A variant allele [22]. Regarding *GSTO2* rs156697 polymorphism, which causes an asparagine to aspartate substitution in amino acid 142 (* Asn142Asp), a strong association between variant GSTO2*G allele and lower *GSTO2* gene expression has been shown [26,27]. These GSTO polymorphisms were independent predictors of a higher risk of death among patients with muscle invasive bladder cancer [28].

Regarding GST omega class polymorphisms in ccRCC, haplotype comprised of all three variant alleles (GSTO1*A (rs4925), GSTO2*G (rs156697) and GSTO2*G (rs2297235)) showed significantly higher risk of disease development compared to haplotype consisting of all three referent alleles [29]. However, their potential functional significance in terms of ccRCC prognosis have not been studied, as yet. Therefore, we aimed to evaluate the effect of specific GSTO gene variants on the postoperative prognosis in patients with ccRCC. Furthermore, we evaluated GSTO1-1 and GSTO2-2 expression in ccRCC and adjacent non-tumor tissue, as well as phosphorylation status of two important survival pathways, PI3K/Akt/mTOR and Raf/MEK/ERK. Potential association of GSTO1-1 with downstream effectors of these signaling pathways was also investigated.

## 2. Results

### 2.1. The Relevance of GSTO1 and GSTO2 Polymorphisms in Overall Survival of ccRCC Patients

The effect of *GSTO1* and *GSTO2* polymorphisms on overall survival was investigated in 228 ccRCC patients during median follow-up period of 67 months ranging from 1 to 153 months. Considering the higher prevalence of disease in men, the overall survival was also studied in male and female subpopulations separately. As presented in Table 1, tumor grade II was shown to be the most frequent among ccRCC patients (G2, 55%). Regarding pT stage, the majority of patients had pT1 and pT3 tumors (45% and 42%, respectively). Similar distribution is observed among male patients (Table 1).

**Figure 1:**
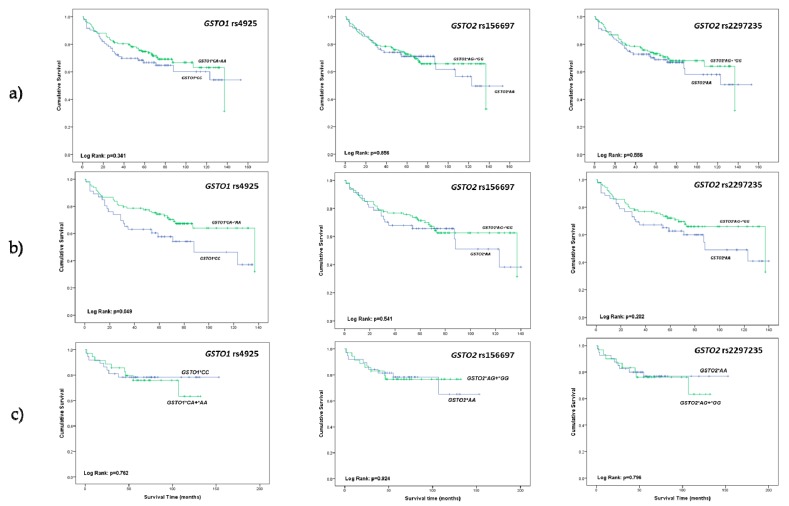
(**a**) Overall survival of clear cell renal cell carcinoma (ccRCC) patients stratified by *GSTO1* (rs4925) and *GSTO2* (rs156697 and rs2297235) polymorphisms; (**b**) overall survival of male ccRCC patients stratified by *GSTO1* (rs4925) and *GSTO2* (rs156697 and rs2297235) polymorphisms; (**c**) overall survival of female ccRCC patients stratified by *GSTO1* (rs4925) and *GSTO2* (rs156697 and rs2297235) polymorphisms.

Among 228 ccRCC patients with successfully acquired follow-up data there were 79 (35%) deaths throughout the follow-up period. Kaplan-Meier survival analysis did not show statistically significant effect of either *GSTO1* (rs4925) or *GSTO2* (rs156697 and rs2297235) polymorphisms on overall survival among ccRCC patients (Figure 1a). Considering the higher propensity to RCC among men, we further focused on estimation of the potential effect of different GSTO genotypes on overall survival in male and female ccRCC patients separately. There were 61 (40%) deaths among 154 men and 18 (24%) deaths among 74 women during the follow-up period. Kaplan-Meier survival analysis demonstrated statistically significant shorter overall survival (log-rank: *p* = 0.049) in male carriers of GSTO1*C/C wild-type genotype in comparison with the male carriers of at least one variant allele (Figure 1b). However, *GSTO2* rs156697 and rs2297235 polymorphisms did not show effect on overall survival among male ccRCC patients (Figure 1b). Furthermore, our results did not exhibit effect of either *GSTO1* (rs4925) or *GSTO2* (rs156697 and rs2297235) polymorphisms on overall survival among female ccRCC patients (Figure 1c).

### 2.2. Predicting Effect of GSTO1 and GSTO2 Polymorphisms on Overall Mortality in ccRCC Patients

The multivariate Cox regression analysis did not show statistically significant association between either *GSTO1* (rs1495) or *GSTO2* (rs156697 and rs2297235) genotypes and overall mortality, adjusted by recognized prognostic factors, Fuhrman nuclear grade and pT stage, among ccRCC patients (Table 2).

However, when we analyzed this association among male patients, the multivariate Cox regression analysis confirmed GSTO1*CC genotype as an independent predictor of higher risk for overall mortality in those patients. Namely, male carriers of GSTO1*CC genotype had an almost two-fold higher mortality risk compared to the carriers of GSTO1*A variant allele (HR = 1.89, 95%CI: 1.04–3.42, *p* = 0.037). Regarding both investigated GSTO2 polymorphisms (rs156697 and rs2297235), the results did not reach statistical significance (*p* > 0.05, Table 3).

The analysis of overall mortality did not show statistically significant association between either investigated GSTO genotypes among female ccRCC patients (*p* > 0.05, Table 4).

### 2.3. Expression of Glutathione Transferase Omega Class Enzymes and Downstream Effectors of PI3K/Akt and Raf/MEK/ERK Signaling Pathway in ccRCC

Tumor and adjacent non-tumor tissue samples were acquired during total nephrectomy from 30 patients with ccRCC. All tumor samples were stratified by their pT stage to early-stage (pT1 and pT2) and late-stage (pT3 and pT4) ccRCC. Cytocolic fractions were used for determination of protein expression and immunoprecipitation analysis.

**Figure 2:**
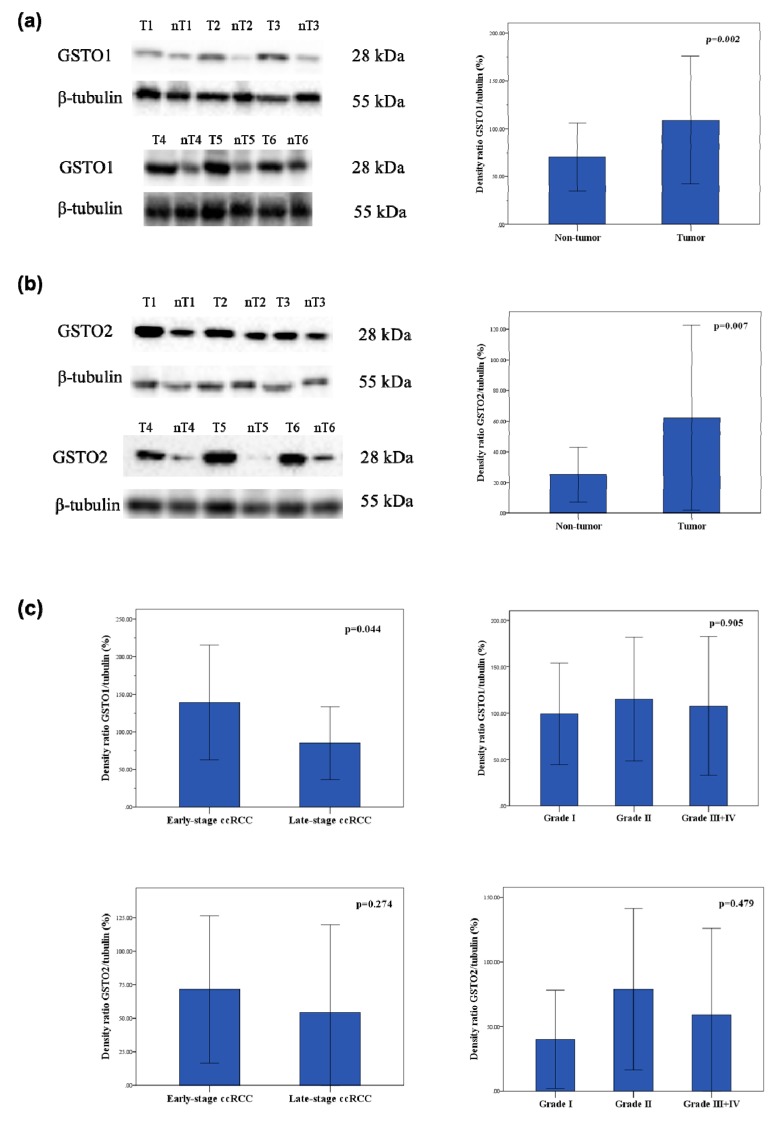

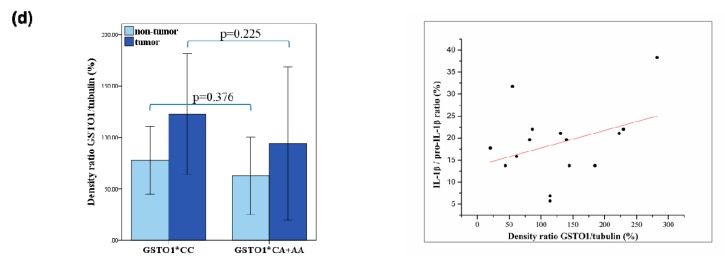
(**a**) Expression of GSTO1 (28 kDa) in ccRCC tumor (T) and corresponding non-tumor (nT) tissue samples; (**b**) expression of GSTO2 (28 kDa) in ccRCC tumor (T) and corresponding non-tumor (nT) tissue samples; (**c**) expression of GSTO1 and GSTO2 (28 kDa) in tumor ccRCC tissue samples according to pT stage and Fuhrman nuclear grade of ccRCC; early-stage ccRCC- pT1 and pT2; late-stage ccRCC- pT3 and pT4; (**d**) expression of GSTO1 stratified according to *GSTO1* polymorphism; correlation between GSTO1 and IL-1β/ pro-IL-1β ratio in tumor ccRCC tissue samples.

Densitometry analysis following Western blot showed 1.5-fold higher expression of GSTO1 in tumor ccRCC compared to non-tumor tissue (*p* = 0.002, Figure 2a, Figure S1, Table S1). Likewise, 2.2-fold higher expression of GSTO2 protein in tumor ccRCC in comparison with non-tumor tissue was found (*p* = 0.007, Figure 2b, Figure S1, Table S1). Since β-actin has been identified as a target for deglutathionylation by GSTO1-1, we used tubulin as a loading control. Representative blots demonstrating higher expression of GSTO1 and GSTO2 in tumor samples compared to respective non-tumor samples are presented in the Figure 2a,b.

Moreover, in ccRCC samples categorized by their pT stage, statistically significant decrease of GSTO1 expression in the late-stage compared to early-stage of disease was found (*p* = 0.044, Figure 2c, Figure S1, Table S1). Similarly, GSTO2 expression was decreased in the late-stage compared to early-stage ccRCC, although without statistical significance (*p* = 0.274, Figure 2c, Figure S1, Table S1). On the contrary, no statistical significance regarding GSTO1 and GSTO2 expression was observed when stratified according to Fuhrman nuclear grade (Figure 2c). Interestingly, in tumor specimens of GSTO1*CC wild-type genotype carriers, higher GSTO1 expression was found in comparison with those carrying at least one variant allele. However, this difference did not reach statistical significance (*p* = 0.225, Figure 2d, Figure S1, Table S1). Considering the role of GSTO1 in modulation of posttranslational processing of IL1-β, we determined the levels of pro-IL-1β and IL-1β in ccRCC tissue. We evaluated correlation between GSTO1 expression and IL-1β/pro-IL-1β ratio in tumor ccRCC tissue samples and observed weak positive correlation (*r* = 0.260, *p* = 0.350) (Figure 2d).

**Figure 3:**
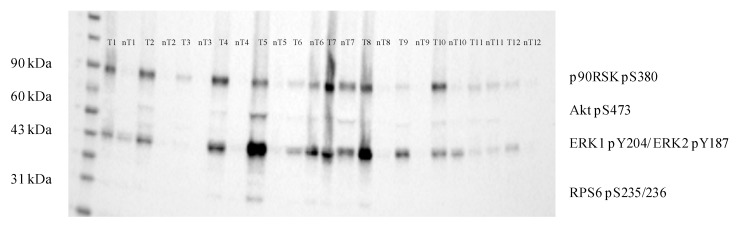
Phosphorylation status of downstream effectors of PI3K/Akt/mTOR and Raf/MEK/ERK signaling pathways in ccRCC tumor (T) and corresponding non-tumor (nT) tissue samples; RSK1p90-90 kDa ribosomal protein S6 kinase 1; Akt—protein kinase B; ERK—extracellular signal-regulated kinase; RPS6—ribosomal protein S6.

Considering the important role of PI3K/Akt/mTOR and Raf/MEK/ERK signaling pathways in ccRCC, we assessed the phosphorylation status of their downstream effectors. Downstream effectors of PI3K/Akt/mTOR pathway, targeted by the antibody cocktail used in Western blot analysis, comprise Akt1 phospho-S473 and ribosomal protein S6 (RPS6) phospho-S235/236, as well as ERK1/2 phospho-Y204/197 and ribosomal protein S6 kinase p90RSK phospho-S380 of Raf/MEK/ERK pathway. Increased expression of RSK1p90 phospho-S380 (*p* = 0.010), Akt1 phospho-S473 (*p* = 0.026) and ERK1/2 phospho-Y204/197 (*p* = 0.015) in tumor ccRCC compared to respective non-tumor tissue has been shown (Figure 3, Figure S1, Table S1).

### 2.4. Immunoprecipitation of GSTO1 and Associated Proteins in Tumor ccRCC Tissue

**Figure 4:**
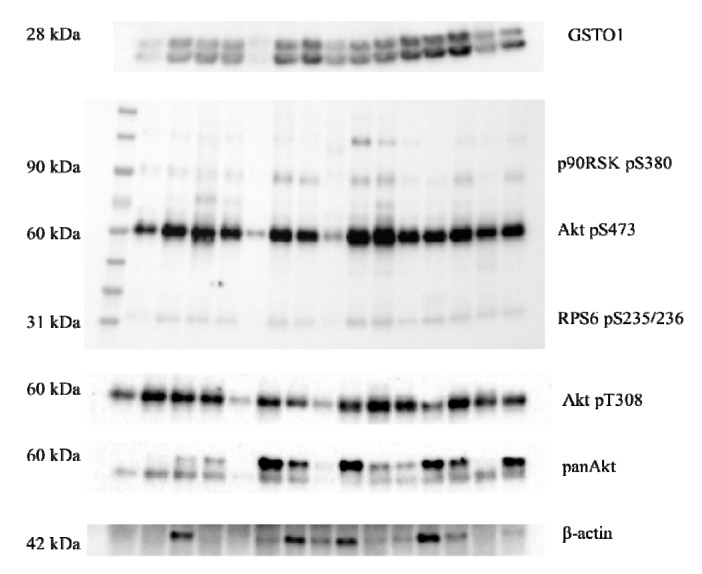
Immunoprecipitation of GSTO1 and associated proteins in tumor ccRCC tissue samples; RSK1p90- 90 kDa ribosomal protein S6 kinase 1; Akt—protein kinase B; RPS6—ribosomal protein S6. Their cytosolic expression was shown in Figure 3.

Furthermore, we investigated potential association of GSTO1 with signaling molecules of PI3K/Akt/mTOR and Raf/MEK/ERK pathways, shown to be upregulated in ccRCC tissue (Figure 3). Western blot analysis, following protein immunoprecipitation of ccRCC tissue samples by anti-GSTO1 antibody, showed association of GSTO1 with RPS6 phospho-S235/236 and Akt phospho-S473, downstream effectors of PI3K/Akt/mTOR pathway (Figure 4, Figure S1). Interestingly, regarding downstream effectors of Raf/MEK/ERK pathway, ERK1/2 phospho-Y204/197 was not co-immunoprecipitated with GSTO1, in contrast to RSK1p90 phospho-S380. Although increased expression of ERK1/2 phospho-Y204/197 was found in tumor ccRCC tissue (Figure 3), the association of this protein with GSTO1 was not observed. Moreover, we demonstrated association of GSTO1 with Akt phospho-T308 and total Akt (panAkt) (Figure 4, Figure S1). Additionally, β-actin co-immunoprecipitated with GSTO1 (Figure 4, Figure S1) as expected considering that β-actin is the target for GSTO1-mediated deglutathionylation.

## 3. Discussion

The results of this study, for the first time, demonstrated statistically significant shorter survival in male carriers of GSTO1*C/C wild-type genotype compared to the carriers of at least one variant allele. In addition, GSTO1*C/C genotype independently predicted higher risk of overall mortality among male ccRCC patients when the association between different gene variants and overall mortality, adjusted by established prognostic factors, was analyzed. In ccRCC tumor tissue, in comparison with the corresponding non-tumor tissue, increased expression of both GSTO1 and GSTO2 was determined. Moreover, interaction of GSTO1 with activated downstream effectors of PI3K/Akt/mTOR and Raf/MEK/ERK pathway was shown in ccRCC tumor tissue.

Analysis of the role of investigated polymorphisms as determinants of postoperative prognosis and the risk of overall mortality in the whole group of ccRCC patients, regardless of gender, did not show a statistically significant effect. Considering that men are predominantly affected by the disease and slightly different modulating effect of risk factors in terms of gender [1], it is plausible that some mechanisms underlying ccRCC progression might also be different. Namely, in many cancers sex-biased disparities have been determined [30]. Until now, increased BMI and hypertension have been shown to increase the long-term RCC risk in men, whereas central adiposity and the waist to hip ratio were positively associated with disease risk in women [1]. Accumulating evidence also indicates that certain genes and proteins that are differentially expressed in male and female ccRCC might further influence sex-biased differences regarding tumor progression. Thus, upregulation of phosphoribosylanthranilate isomerase 1 (PAI-1), protein kinase C alpha (PKC-alpha), vascular endothelial growth factor receptor 2 (VEGFR2), androgen receptor (AR), Ras homologue gene family member J (ARHJ), insulin receptor substrate 1 (IRS1) and C-Jun activation domain-binding protein-1 (JAB1) genes was found in males with ccRCC, while increased expression of N-myc downstream regulated 1 (NDRG1), Akt, phosphatase and tensin homolog (PTEN), DJ-1, 4E binding protein 1 (4E-BP1), Src and p38 was shown in females [30]. Our results on different prognostic significance of GSTO1 polymorphism in male and female patients with ccRCC is a further proof of gender-specific molecular patterns in ccRCC. Exploring molecular mechanisms of the gender effect on cancer progression is important, especially regarding the monitoring of high risk patients. Possible underlying mechanism of prognostic significance of *GSTO1* polymorphism in male ccRCC might be the role of GSTO1 in IL-1β posttranslational processing [20]. Indeed, literature data indicate that GSTO1 might be a component of the inflammasomes, multi-protein complexes regulating the production of this pro-inflammatory cytokine [31]. Petrella and Vincenti elucidated that previously established association between high levels of IL-1β and RCC progression [32] might be explained by the stimulation of tumor cell invasion. Namely, IL-1β induces the expression of matrix metaloproteinases (MMP) through the activation of the transcription factor CCAAT enhancer binding protein β (CEBP β) [33]. Considering that GSTO1-1 activity is affected by GSTO1 allelic variant, it seems plausible that GSTO1 polymorphism might also influence activation of IL-1β. Considering recognized variance in immune response capacity of males and females, it might be hypothesized that one of molecular mechanisms involved in gender-specific survival difference observed in our study is contributing role of GSTO1-1 in inflammation [20,34].

We conducted additional investigations to elucidate the potential molecular mechanisms underlying the GSTO contribution to ccRCC progression. Additionally, to clarify the clinical meaning of *GSTO1* polymorphism, we assessed the expression of GSTO1 in relation to SNP status. Moreover, phosphorylation status of two constitutively active survival pathways in ccRCC, PI3K/Akt and MAPK/ERK, which potentiate cell growth, proliferation and invasion in cancer, were also studied [6].

Our results showed significantly higher protein expression of GSTO1 and GSTO2 in tumor ccRCC tissue compared to non-tumor tissue. We found slightly different GSTO1 expression in relation to its most investigated polymorphism, which is in concordance with the study of Mukherjee et al. investigating functional implications of GSTO1 variants [27]. Moreover, decrease of GSTO1 and GSTO2 expression in the late-stage compared to early-stage ccRCC was observed. However, the change between different stages of disease did not reach statistical significance regarding GSTO2 expression. The GSTO1 upregulation has been reported in different cancers, including bladder [35], pancreatic, ovarian cancer [24,36] and esophageal adenocarcinoma [37]. Moreover, nuclear localization of GSTO1 in Barrett’s esophagus [38] and colorectal carcinoma [39] suggests its probable involvement in the protection of specific nuclear components in conditions of oxidative stress, thus promoting malignant transformation. Unfortunately, there are no literature data on GSTO2 expression in cancer, as yet.

Significant change between early-stage and late-stage ccRCC regarding GSTO1 expression levels might be explained by complex changes of redox homeostasis throughout ccRCC progression [40]. Modification of cancer cells’ metabolic phenotype enables maintenance of high ROS levels within a narrow range, allowing them to enhance growth and invasion, together with limitation of their apoptotic potential [41]. Distinctive for RCC is the shift in ratio of reduced and oxidized form of glutathione (GSH/GSSG) between early- and late-stage of disease, which is accompanied by the decrease of enzymes involved in GSH metabolism only in the early-stage RCC [40,42]. It could be speculated that GSTO1-1 affects progression of ccRCC by at least two mechanisms. Namely, higher deglutathionylase activity might increase exposure of proteins to oxidative modifications in early-stage ccRCC. Additionally, deglutathionylation seem to regulate and modulate function of the affected proteins. Considering the role of GSTO1-1 in glutathionylation cycle [22], it can be assumed that increased GSTO1-1 expression in ccRCC might be involved in regulation of redox-sensitive signaling pathways by its deglutathionylase activity. The relevance of glutathionylation status in redox signaling regulation was confirmed in investigations concerning glutaredoxins, as the major intracellular enzymes with deglutathionylase activity [22]. Numerous proteins involved in signaling (kinases and phosphatases), protein folding and stability, redox homeostasis, calcium homeostasis, energy metabolism and glycolysis, as well as cytoskeletal proteins, transcription factors and heat shock proteins are regulated via S-glutathionylation [43,44]. β-actin, heat shock protein 70, heat shock protein 7c and prolactin-inducible protein have been identified as targets for deglutathionylation by GSTO1-1 [22]. In accordance with this are our results showing co-immunoprecipitation of β-actin with GSTO1.

Furthermore, our study showed a weak correlation between IL-1β/pro- IL-1β ratio, as a degree of IL-1β activation, and the level of GSTO1 expression in ccRCC tumor tissue. It might be speculated that this result reflects the established role of GSTO1-1 in modulation of IL-1β posttranslational processing [20]. However, for the potential assessment of stronger correlation, a larger study would be warranted.

The PI3K/Akt signaling pathway, involved in the regulation of proliferation, differentiation and survival of cancer cells, is highly activated in ccRCC [45]. It has been shown that the phosphorylation of Akt at S473 (Akt1 pS473), either by mTOR or by DNA-dependent protein kinase, promotes complete enzymatic activity of Akt [46]. Numerous downstream Akt effectors implicated in ccRCC progression include the mammalian target of rapamycin (mTOR), glycogen synthase kinase 3, Bcl-2-associated death promoter, NF-κB, as well as MAPK pathways signaling molecules, c-Jun NH2-terminal kinase (JNK) and extracellular signal-regulated kinase (ERK) [45]. Furthermore, among mTOR complex 1 (mTORC1) substrates involved in promoting cellular proliferation is ribosomal protein S6 kinase (S6K1/RSK1), which phosphorylates the ribosomal protein S6 (S6/RPS6) [46]. The results of this study demonstrated the increased expression of activated downstream effectors of PI3K/Akt/mTOR and Raf/MEK/ERK pathway in ccRCC tumor tissue compared to adjacent non-tumor tissue. These results confirmed constitutive activation of two important pro-survival pathways in ccRCC.

Further investigations were focused on the possible association of GSTO1 with analyzed signaling molecules. Based on data on co-immunoprecipitation of GSTO1 with Akt and several other downstream signaling molecules we assumed that those proteins might be the targets for GSTO1-mediated deglutathionylation. Indeed, a deglutathionylation modification was implicated in the activation of Akt and RSK1, downstream effector of mTOR pathway [8,47]. The possible regulation of Akt and RSK1 activity by GSTO1-1 might be of importance in different cellular processes, including survival, growth, proliferation and metabolism [46]. Interestingly, we did not find association of GSTO1 with ERK1/2. Since there is no evidence that ERK1/2 can be regulated by glutathionylation, further investigations would be necessary.

Several molecular targets of the PI3K/Akt signaling cascade have been suggested in antitumor therapy [45]. Regarding metastatic ccRCC, several targeted therapies against VEGF have been approved, including sorafenib, sunitinib, pazopanib and axitinib [1]. Recently, it has been shown that some of these drugs also affect cellular redox homeostasis, beside their primary antitumor role [48]. In contrast to sorafenib, which shows prooxidant effects by a decrease in GSH level [49], sunitinib exhibits antioxidant effects via increase in GSH level and inhibition of neuronal nitric oxide synthase activity (NOS) [50]. Considering influence of those targeted therapies on redox state, research concerning GSTO1 inhibitors in cancers could be valuable. Several studies suggested promising results on antitumor effect of α-chloroacetamide-1, highly specific and highly sensitive GSTO1 inhibitors [51]. Namely, KT53 initiated a significant increase in cisplatin-induced cell death in the human breast cancer cell line [51]. Additionally, another member of this class of inhibitors, C1-27 showed potent antitumor activity in colorectal cancer cell lines and in vivo models of disease [52].

Further investigations clarifying relevance of GSTO1 and GSTO2 expression pattern during ccRCC progression could be valuable. Considering the relevance of investigated signaling pathways in progression of ccRCC, future research directions could be focused on determination of GSTO1 expression in metastatic disease, as well as its potential as a therapy target. In view of interaction between GSTO1 and investigated signaling molecules involved in cell survival, it would be of great importance to investigate effect of GSTO1 specific inhibitors on signaling pathways, as well as different cell death modalities on ccRCC cell lines. Beside the results on the effect of GSTO polymorphisms on overall survival, it would be beneficial to investigate its potential association with cancer specific survival in a larger cohort. Moreover, future studies on combined effect of several different GSTO1 polymorphisms on prognosis of ccRCC patients could be valuable.

## 4. Materials and Methods

### 4.1. Study Population

In this study, 239 patients (162 men, 77 women; average age 58.94 ± 11.64 years) from the Clinic of Urology, Clinical Center of Serbia, Belgrade, diagnosed with ccRCC were enrolled to investigate the prognostic significance of GSTO polymorphisms in ccRCC. Fuhrman nuclear grade and stage of each tumor was acquired by histopathological examination, according to Srigley et al. [53] and Tumor Node Metastais classification by Sobin et al. [54]. The follow-up information was available for 228 patients (154 men, 74 women; average age 59.19 ± 11.58). Thirty tumor and corresponding non-tumor tissue samples were obtained from patients that have undergone radical nephrectomy. The study was approved by the Institutional Ethical board (13 October 2011, approval number 29/X-3, Faculty of Medicine, University of Belgrade, Serbia and 6 July 2017, approval number 29/VII-14) and performed in accordance with ethical principles of the World Medical Association Declaration of Helsinki. Informed written consent was obtained from all patients.

### 4.2. Sample Preparation

Genomic DNA was isolated by QIAamp DNA Blood Mini Kit (Qiagen, Hilden, Germany) from 239 blood samples according to manufacturer’s instructions. Cytosolic fractions of ccRCC and adjacent non-tumor tissue specimen were obtained after homogenization in lysis buffer (50 mmol/L Tris, 200 mmol/L NaCl, 1 mmol/L dithiothreitol, pH 7.8) supplemented with protease and phosphatase inhibitors (Sigma-Aldrich, St. Louis, MO, USA) for determination of protein expression and IL-1β level.

### 4.3. Genotyping

Genotyping of GSTO1*C419A (rs4925), GSTO2*A424G (rs156697) and GSTO2*A183G (rs2297235) polymorphisms was achieved by quantitative polymerase chain reaction (qPCR) on Mastercycler ep realplex (Eppendorf, Hamburg, Germany) using TaqMan SNP Genotyping assays (Thermo Fisher Scientific, Waltham, MA, USA, assay ID: C_11309430_30, C_3223136_1, C_3223142_1, respectively).

### 4.4. Western Blot and Immunoprecipitation

Equal amounts of proteins (30 μg) from acquired ccRCC and corresponding non-tumor cytosolic fractions were subjected to SDS-PAGE on Criterion™ TGX precast 26-well gels (4–15%) (Bio-Rad, Hercules, CA, USA) followed by the transfer of proteins onto nitrocellulose membrane. Immunodetection of proteins transferred to membrane was conducted by using primary antibodies against GSTO1 (mouse polyclonal Abcam, Cambridge, UK), GSTO2 (rabbit polyclonal, GeneTex, Irvine, CA, USA), Akt (Cell Signaling, Danvers, MA, USA), phospho-Akt (T308) (rabbit monoclonal, Cell Signaling, USA) and β-tubulin (mouse monoclonal, Sigma-Aldrich, USA). Akt/MAPK Signaling Pathway Antibody Cocktail was used for simultaneous detection of phosphorylated 90kDa ribosomal protein S6 kinase 1 (RSK1p90) phospho-S380, protein kinase B (Akt) phospho-S473, extracellular-signal-regulated kinase (ERK1 phospho-Y204)/ERK2 phospho-Y187) and ribosomal protein S6 (RPS6) phospho-S235/236 (rabbit, Abcam, UK). The next step was the incubation of membranes with appropriate HRP-conjugated secondary antibodies (anti-mouse developed in goat, Abcam, Cambridge, UK; anti-rabbit developed in donkey, GE Healthcare, UK). For visualization, Clarity™ Western ECL Substrate (Bio-Rad, USA) was used, followed by detection of chemiluminescence on ChemiDoc™ MP Imaging System (Bio-Rad, USA). ImageLab software (Bio-Rad, USA) was used for densitometric analysis of obtained blots.

Immunoprecipitation was performed using Catch and Release^®^ v2.0 High Throughput (HT) Immunoprecipitation Assay Kit (Merck Millipore, Germany). ccRCC cytosolic fractions were incubated with the antibody against GSTO1 (Abcam, UK). Immunoprecipitated proteins were subjected to electrophoresis and Western blot analysis, as previously described.

### 4.5. Determination of IL-1β and pro- IL-1β Levels

The quantification of interleukin-1β (IL-1β) in ccRCC cytosolic fractions was assessed by Platinum ELISA (enzyme-linked immunosorbent assay) kit (Affimetrix, eBioscience, San Diego CA, USA), whereas the quantitative detection of pro-interleukin-1β (pro-IL-1β) was performed using Human pro-IL-1β ELISA kit (Elabscience Biotechnology Inc, Houston, TX, USA) according to the manufacturer’s instructions.

### 4.6. Statistical Analysis

Statistical analysis was performed by Statistical Package for the Social Sciences (SPSS software version 17, SPSS Inc, Chicago, IL, USA). Kaplan-Meier analysis was used to evaluate the effect of GSTO genotypes on overall survival of ccRCC patients. Survival time was estimated as time from nephrectomy to the date of death or last follow-up (1 March 2018). The follow-up information was attainable for 228 ccRCC patients owing to the loss of 11 patients’ contact information. Median follow-up was 67 months, ranging from 1 to 153 months. The long-rank test was used for the evaluation of differences in survival between different genotypes of each polymorphism. The prognostic value of investigated polymorphisms in overall mortality was estimated by the Cox regression analysis, adjusted by Fuhrman nuclear grade and pT stage, as established prognostic factors.

The difference in expression of investigated proteins in tumor compared to respective non-tumor tissue was evaluated by Wilcoxon test, whereas expression of GSTO1 and GSTO2 stratified by pT stage and Fuhrman nuclear grade was analyzed using Mann–Whitney rank-sum test and Kruskal-Wallis test, respectively. The association between GSTO1 expression and IL-1β/pro-IL1β ratio was determined using Spearman’s coefficient of linear correlation.

A *p* value of ≤0.05 was considered to be statistically significant.

## 5. Conclusions

This study demonstrated a significant prognostic role of GSTO1 polymorphism in ccRCC. Furthermore, up-regulated GSTO1-1 and GSTO2-2 enzymes in ccRCC tumor tissue might contribute to aberrant redox homeostasis. The possible molecular mechanism underlying the role of GSTO1-1 in ccRCC progression might be partially explained by its deglutathionylase activity.

## Figures and Tables

**Table 1:** Clinicopathological characteristics of the 228 patients with ccRCC.

Characteristics	Patients, *n* (%)	Male Patients, *n* (%)
Age, years (mean ± SD)	59.19 ± 11.58	57.79 ± 11.22
Gender, *n* (%)		
Female	74 (32)	
Male	154 (68)	
Fuhrman nuclear grade, *n* (%)		
G1	28 (14)	17 (12)
G2	111 (56)	76 (57)
G3	50 (26)	36 (27)
G4	8 (4)	5 (4)
pT stage, *n* (%)		
pT1	97 (45)	61 (40)
pT2	24 (11)	14 (10)
pT3	90 (42)	69 (47)
pT4	5 (2)	4 (3)

**Table 2:** Predicting effect of glutathione transferase omega (GSTO) polymorphisms on overall mortality in ccRCC patients.

Variable	Category	Events, *n* (%)	HR (95% CI) ^b^	*p*
***GSTO1* rs4925**				
FNR ^a^	G1/G2/G3/G4	2 (7)/32 (29)/29 (58)/5 (63)	1.57 (1.08–2.27)	0.017
pT stage	pT1/pT2/pT3/pT4	14 (14)/8 (33)/ 51 (57)/3 (60)	2.01 (1.46–2.76)	<0.001
*GSTO1* rs4925				
*CC		32 (38)	1.53 (0.91–2.58)	0.107
*CA + *AA		47 (34)	1.00	
***GSTO2* rs156697**				
FNR	G1/G2/G3/G4	2 (7)/32 (29)/29 (58)/5 (63)	1.58 (1.09–2.27)	0.015
pT stage	pT1/pT2/pT3/pT4	14 (14)/8 (33)/51 (57)/3 (60)	1.97 (1.43–2.70)	<0.001
*GSTO2* rs156697				
*AA		31 (35)	1.11 (0.66–1.88)	0.689
*AG + *GG		48 (34)	1.00	
***GSTO2* rs2297235**				
FNR	G1/G2/G3/G4	2 (7)/32 (29)/29 (58)/5 (63)	1.57 (1.08–2.27)	0.016
pT stage	pT1/pT2/pT3/pT4	14 (14)/8 (33)/51 (57)/3 (60)	1.96 (1.43–2.69)	<0.001
*GSTO2* rs2297235				
*AA		34 (36)	1.24 (0.74–2.07)	0.425
*AG + *GG		44 (34)	1.00	

^a^ Fuhrman nuclear grade; ^b^ HR, odds ratio adjusted to Fuhrman nuclear grade and pT stage; CI, confidence interval; *p* < 0.05 was considered to be statistically significant.

**Table 3:** Predicting effect of GSTO polymorphisms on overall mortality in male ccRCC patients.

Variable	Category	Events, *n* (%)	HR (95% CI) ^b^	*p*
***GSTO1* rs4925**				
FNR ^a^	G1/G2/G3/G4	2 (12)/23 (30)/26 (72)/2 (40)	1.58 (1.03–2.43)	0.037
pT stage	pT1/pT2/pT3/pT4	10(16)/5 (36)/42 (61)/2 (50)	1.83 (1.28–2.62)	0.001
*GSTO1* rs4925				
*CC		23 (49)	1.89 (1.04–3.42)	0.037
*CA + *AA		38 (36)	1.00	
***GSTO2* rs156697**				
FNR	G1/G2/G3/G4	2 (12)/23 (30)/26 (72)/2 (40)	1.56 (1.02–2.38)	0.040
pT stage	pT1/pT2/pT3/pT4	10(16)/5 (36)/42 (61)/2 (50)	1.83 (1.29–2.60)	0.001
*GSTO2* rs156697				
*AA		21 (43)	1.32 (0.71–2.43)	0.380
*AG + *GG		40 (38)	1.00	
***GSTO2* rs2297235**				
FNR	G1/G2/G3/G4	2 (12)/23 (30)/26 (72)/2 (40)	1.59 (1.04–2.46)	0.034
pT stage	pT1/pT2/pT3/pT4	10(16)/5 (36)/42 (61)/2 (50)	1.81 (1.27–2.59)	0.001
*GSTO2* rs2297235				
*AA		24 (45)	1.60 (0.88–2.92)	0.127
*AG + *GG		36 (37)	1.00	

^a^ Fuhrman nuclear grade; ^b^ HR, odds ratio adjusted to Fuhrman nuclear grade and pT stage; CI, confidence interval; *p* < 0.05 was considered to be statistically significant.

**Table 4:** Predicting effect of GSTO polymorphisms on overall mortality in female ccRCC patients.

Variable	Category	Events, *n* (%)	HR (95% CI) ^b^	*p*
***GSTO1* rs4925**				
FNR ^a^	G1/G2/G3/G4	0 (0)/9 (26)/3 (21)/3 (100)	1.92 (0.87–4.26)	0.107
pT stage	pT1/pT2/pT3/pT4	4(11)/3 (30)/9 (43)/1 (100)	2.20 (1.08–4.46)	0.029
*GSTO1* rs4925				
*CC		9 (24)	1.01 (0.34–3.00)	0.992
*CA + *AA		9 (26)	1.00	
***GSTO2* rs156697**				
FNR	G1/G2/G3/G4	0 (0)/9 (26)/3 (21)/3 (100)	1.95 (0.89–4.27)	0.098
pT stage	pT1/pT2/pT3/pT4	4(11)/3 (30)/9 (43)/1 (100)	2.23 (1.11–4.47)	0.024
*GSTO2* rs156697				
*AA		10 (26)	0.78 (0.27–2.28)	0.654
*AG+*GG		8 (23)	1.00	
***GSTO2* rs2297235**				
FNR	G1/G2/G3/G4	0 (0)/9 (26)/3 (21)/3 (100)	1.99 (0.90–4.38)	0.089
pT stage	pT1/pT2/pT3/pT4	4(11)/3 (30)/9 (43)/1 (100)	2.11 (1.05–4.24)	0.035
*GSTO2* rs2297235				
*AA		10 (24)	0.77 (0.26–2.31)	0.645
*AG+*GG		8 (27)	1.00	

^a^ Fuhrman nuclear grade; ^b^ HR, odds ratio adjusted to Fuhrman nuclear grade and pT stage; CI, confidence interval; *p* < 0.05 was considered to be statistically significant.

## Supplementary Materials

The following are available online at https://www.mdpi.com/2072-6694/11/12/2038/s1, Figure S1: Whole Western blots with molecular weights, Table S1: Densitometry readings.
