# Supplementary material for: GSTO1*CC Genotype (rs4925) Predicts Shorter Survival in Clear Cell Renal Cell Carcinoma Male Patients
Source: Cancers (Basel). 2019 Dec 17;11(12):2038. doi: 10.3390/cancers11122038 (PMC6966599; doi:10.3390/cancers11122038)
Supplement: Supplementary file 1 [file cancers-11-02038-s001.zip › cancers-659957-supplementary/Supplemental materials.pdf]

# Supplementary Materials: *GSTO1**CC Genotype (rs4925) Predicts Shorter Survival in Clear Cell Renal Cell Carcinoma Male Patients

**Tanja Radic, Vesna Coric, Zoran Bukumiric, Marija Pljesa-Ercegovac, Tatjana Djukic, Natasa Avramovic, Marija Matic, Smiljana Mihailovic, Dejan Dragicevic, Zoran Dzamic, Tatjana Simic and Ana Savic-Radojevic**

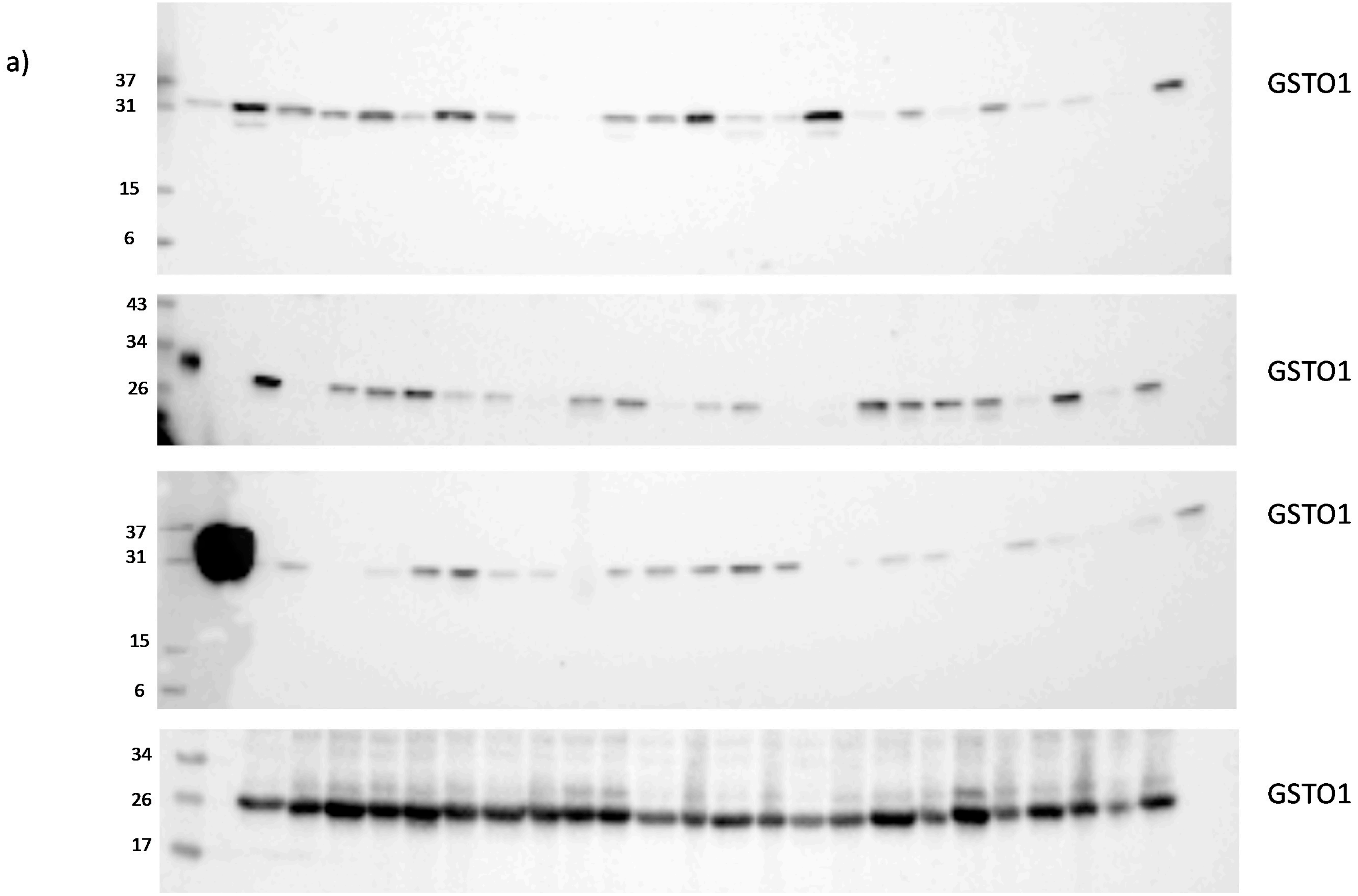
a)
37
31
15
6
GSTO1
43
34
26
GSTO1
37
31
15
6
GSTO1
34
26
17
GSTO1

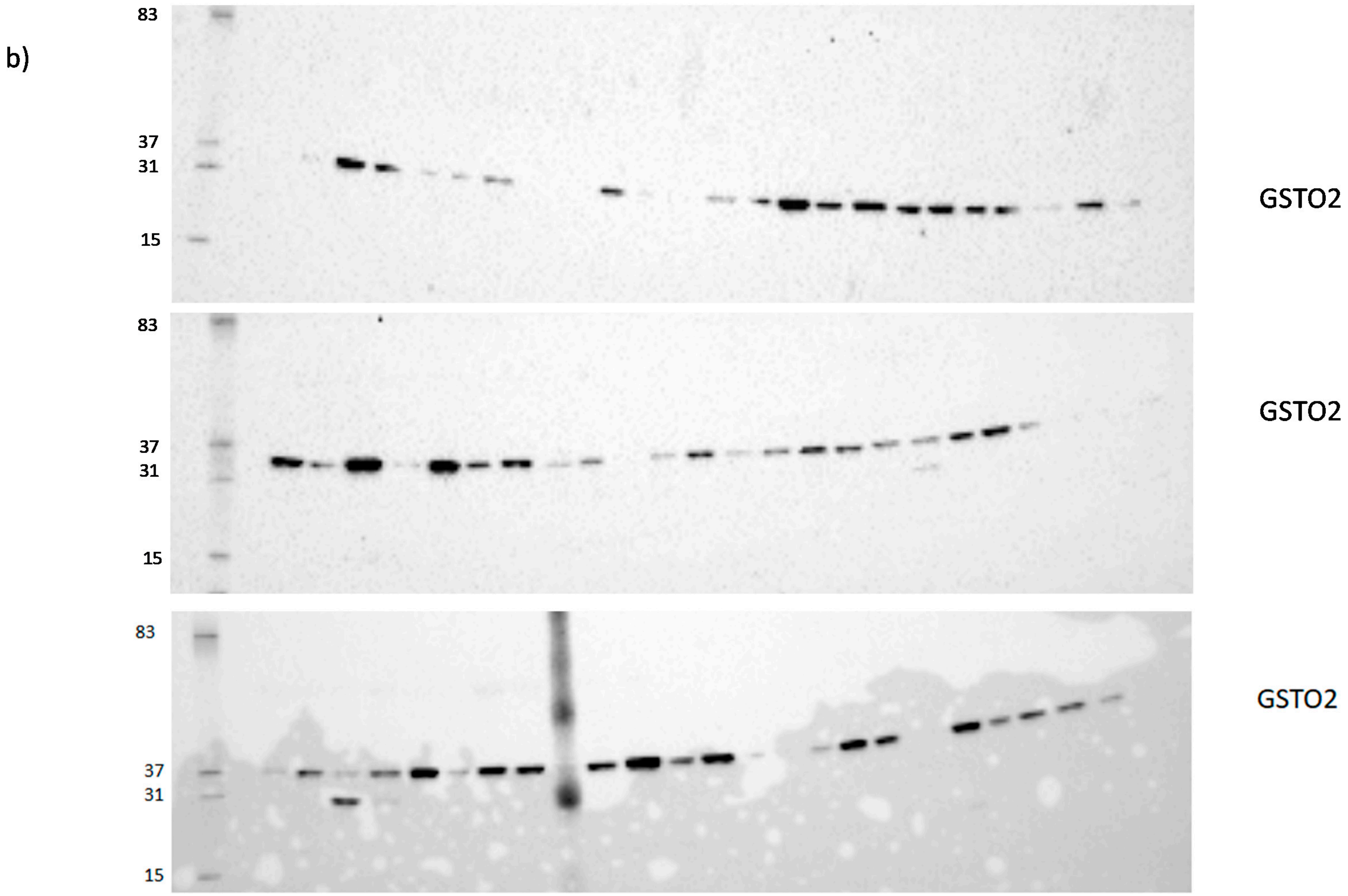

b)
83
37
31
15
GSTO2
83
37
31
15
GSTO2
83
37
31
15
GSTO2

c)

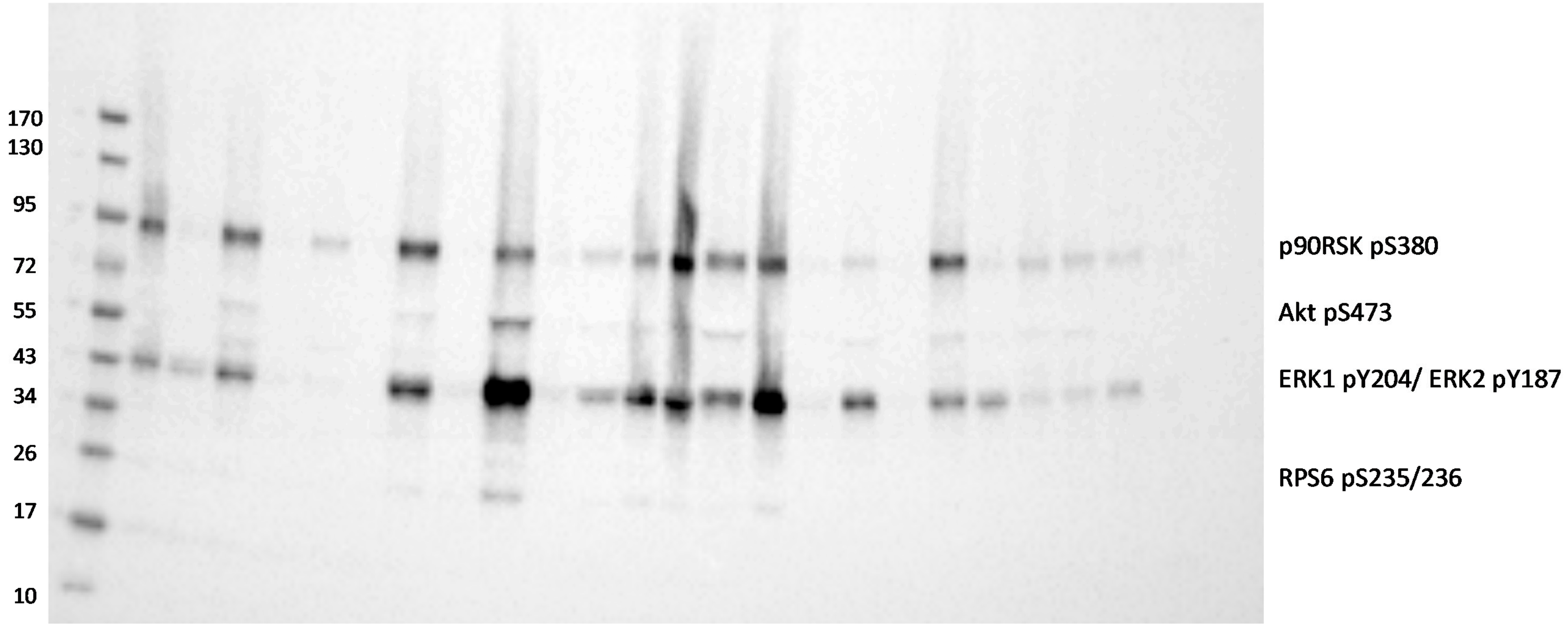
170
130
95
72
55
43
34
26
17
10
p90RSK pS380
Akt pS473
ERK1 pY204/ ERK2 pY187
RPS6 pS235/236

d)

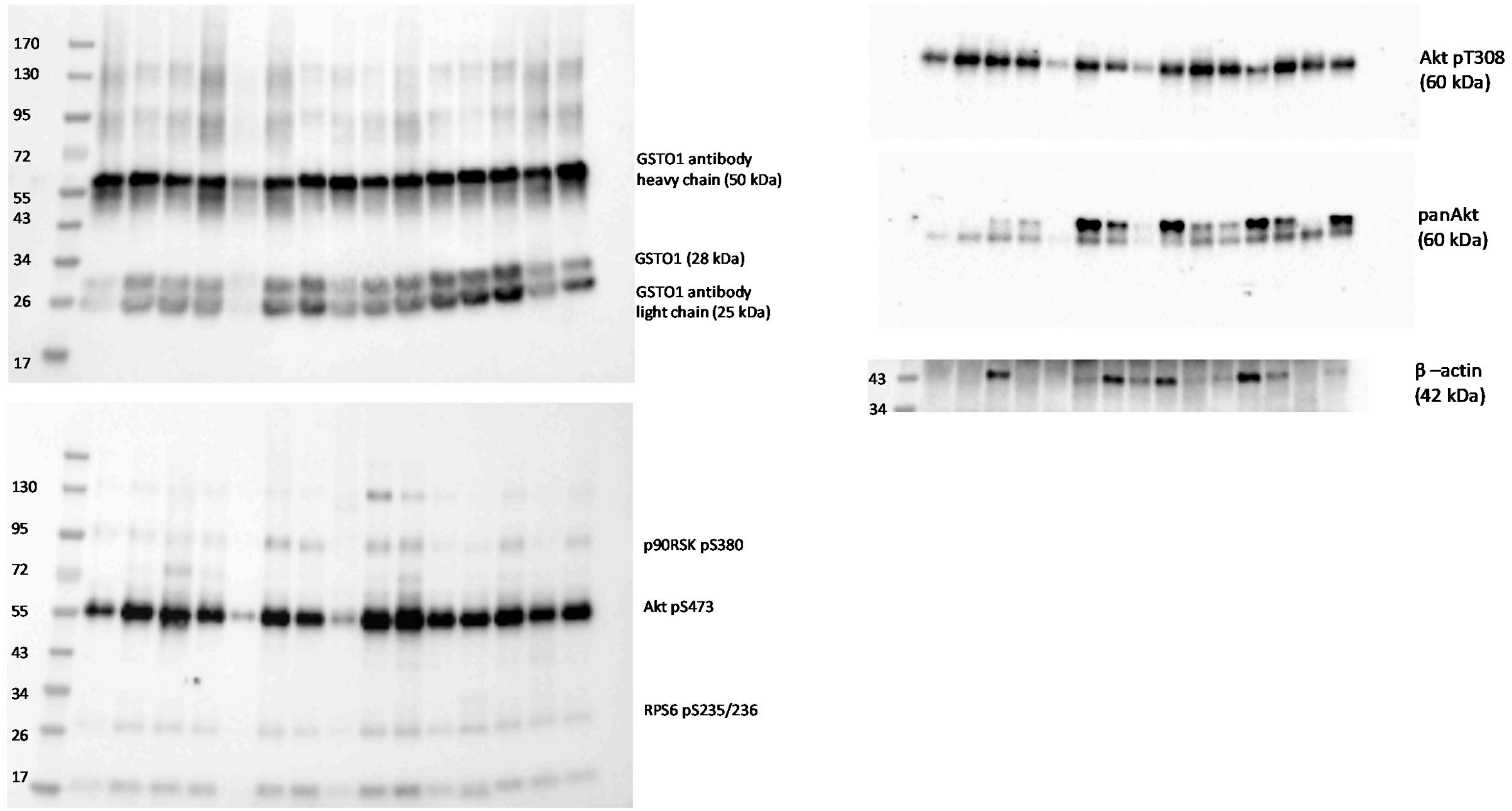


**Figure S1.** Whole Western Blots with molecular weights. (**a**) Western blots for figure 2a). (**b**) Western blots for figure 2b). (**c**) Western blot for figure 3. (**d**) Western blots for figure 4.

© 2019 by the authors. Licensee MDPI, Basel, Switzerland. This article is an open access article distributed under the terms and conditions of the Creative Commons Attribution (CC BY) license (http://creativecommons.org/licenses/by/4.0/).
